# Out-Patient versus In-Patient Arteriovenous Fistula Creation for Dialysis: Assessing Cost-Effectiveness Alongside Clinical Implications

**DOI:** 10.3390/healthcare12111102

**Published:** 2024-05-28

**Authors:** Eliza Russu, Andreea-Cătălina Munteanu, Emil-Marian Arbănași, Ludovic-Alexandru Szanto, Reka Bartus, Elena Florea, Bogdan Corneliu Bandici, Eliza-Mihaela Arbănași, Alexandru Petru Ion, Bogdan Andrei Cordoș, Gabriel Serac, Alexandru-Andrei Ujlaki-Nagi, Claudiu Constantin Ciucanu, Adrian Vasile Mureșan

**Affiliations:** 1 Department of Vascular Surgery, George Emil Palade University of Medicine, Pharmacy, Science and Technology of Targu Mures, 540139 Targu Mures, Romania; eliza.russu@umfst.ro (E.R.); claudio.ciucanu@gmail.com (C.C.C.); adrian.muresan@umfst.ro (A.V.M.); 2 Clinic of Vascular Surgery, Mures County Emergency Hospital, 540136 Targu Mures, Romania; 3 Doctoral School of Medicine and Pharmacy, George Emil Palade University of Medicine, Pharmacy, Science and Technology of Targu Mures, 540139 Targu Mures, Romania; 4 Regenerative Medicine Laboratory, Centre for Advanced Medical and Pharmaceutical Research (CCAMF), George Emil Palade University of Medicine, Pharmacy, Science and Technology of Targu Mures, 540139 Targu Mures, Romania; bogdan.cordos@umfst.ro; 5 George Emil Palade University of Medicine, Pharmacy, Science and Technology of Targu Mures, 540139 Targu Mures, Romania; 6 Centre for Experimental Medical and Imaging Studies, George Emil Palade University of Medicine, Pharmacy, Science and Technology of Targu Mures, 540139 Targu Mures, Romania; 7 Department of Anatomy, George Emil Palade University of Medicine, Pharmacy, Science and Technology of Targu Mures, 540139 Targu Mures, Romania; 8 Psychiatry Clinic 2, Mures County Clinical Hospital, 540139 Targu Mures, Romania

**Keywords:** arteriovenous fistula, vascular access, ambulatory, in-patients, out-patients, vascular surgery

## Abstract

(1) Background: The surgical procedure to create an arteriovenous fistula (AVF) can be performed in either an ambulatory or in-patient hospital setting, depending on the case’s complexity, the anesthesia type used, and the patient’s comorbidities. The main scope of this study is to assess the cost-effectiveness and clinical implications of surgically creating an AVF in both ambulatory and in-hospital settings. (2) Methods: We conducted a retrospective observational study, in which we initially enrolled all patients with end-stage kidney disease (ESKD) admitted to the Vascular Surgery Department, Emergency County Hospital of Targu Mures, Romania, to surgically create an AVF for dialysis, between January 2020 and December 2022. The primary endpoint of this study is to assess the cost-effectiveness of surgically creating an AVF in an ambulatory vs. in-hospital setting by comparing the costs required for the two types of admissions. Further, the 116 patients enrolled in this study were divided into two groups based on their preference for hospitalization: *out-patients* and *in-patients*. (3) Results: Regarding in-patient comorbidities, there was a higher prevalence of peripheral artery disease (PAD) (*p* = 0.006), malignancy (*p* = 0.020), and previous myocardial infarction (*p* = 0.012). In addition, active smoking (*p* = 0.006) and obesity (*p* = 0.018) were more frequent among these patients. Regarding the laboratory data, the in-patients had lower levels of white blood cells (WBC) (*p* = 0.004), neutrophils count (*p* = 0.025), lymphocytes (*p* = 0.034), and monocytes (*p* = 0.032), but there were no differences between the two groups regarding the systemic inflammatory biomarkers or the AVF type. Additionally, we did not register any difference regarding the outcomes: local complications (*p* = 0.588), maturation failure (*p* = 0.267), and primary patency (*p* = 0.834). In our subsequent analysis, we discovered no significant difference between the hospitalization type chosen by patients regarding AVF primary patency failure (*p* = 0.195). We found no significant association between the hospitalization type and the recorded outcomes (all *ps* > 0.05) in both multivariate linear regression and Cox proportional hazard analysis. (4) Conclusions: In conclusion, there are no significant differences in the clinical implications, short-term and long-term complications of AVF for out-patient and in-patient admissions. Additionally, we found no variation in the costs associated with laboratory tests and surgical supplies for an AVF creation. Therefore, it is safe to perform ambulatory AVFs, which can reduce the risk of hospital-acquired infections and provide greater comfort to the patient.

## 1. Introduction

Chronic kidney disease (CKD) is a medical condition that causes a gradual loss of liver function over time. This condition can lead to complications such as fluid retention, electrolyte imbalances, and waste buildup in the body [1,2]. To address the impact of CKD on kidney function, the treatment and management options are tailored to the individual stage of the disease [1,2]. Further, in end-stage kidney disease (ESKD), patients require renal replacement therapy (RRT), such as peritoneal dialysis (PD) or hemodialysis (HD), as their kidneys no longer function adequately [1,2].

The annual report of the European Renal Association—Registry of the European Dialysis and Transplantation Association (ERA-EDTA) revealed that in 2019 [3], there were 893 patients per million population (pmp) who required RRT. This is an increase compared to the previous reports in 2015 [4] and 2016 [5], which recorded a prevalence of 801 pmp and 823 pmp, respectively. Additionally, the reports found an increase in the prevalence of diabetes among dialysis patients between 2015 and 2019 [3,4,5,6]. Moreover, an increase in the prevalence of RRT and diabetes, as well as age, has also been registered at the national level in Romania, according to the same reports [3,4,5,6].

HD is the well-known and most frequently used method of RRT, and to perform it, the patient requires optimal vascular access (VA), which is superficial, easy to puncture, and with good long-term permeability [7,8]. The three types of VA currently used are arteriovenous fistula (AVF), arteriovenous grafts (AVGs), and central venous catheters (CVCs), according to the guidelines of the European Society of Vascular Surgery (ESVS) [9]. An AVF is a connection created surgically between an artery and a vein, which is typically placed at the wrist, forearm, or antecubital fossa [9]. The location for the AVF is chosen based on factors such as vessel size, quality, and the patient’s vascular anatomy [9]. There are various types of AVFs used for HD, but the most common ones are radiocephalic AVF (RC-AVF), brachiocephalic AVF (BC-AVF), and brachiobasilic AVF (BB-AVF) [9,10,11]. However, long-term AVF patency is unsatisfactory due to intimal hyperplasia [12,13]. Moreover, there is an increased risk of complications, such as pseudoaneurysmal development at the puncture site [14,15] and overall aneurysmal development, with a high risk of skin necrosis and rupture [14,15,16,17].

The surgical procedure to create an AVF can be performed in either an ambulatory or in-patient hospital setting, depending on the case’s complexity, anesthesia type used, and the patient’s comorbidities, which may require medical supervision [18,19,20,21,22,23]. More recently, the COVID-19 pandemic significantly impacted medical activity worldwide [24,25]. Patients with ESKD were particularly affected, with an increase in the number of patients requiring dialysis [26], an increase in the frequency of dialysis sessions [26], and a higher demand for AVF surgery [27].

The main scope of this study is to assess the cost-effectiveness and clinical implications of surgically creating an AVF in an ambulatory vs. in-hospital setting. In addition, we will analyze the risk factors that have a predictive role in the short- and long-term failure of primary AVF patency.

## 2. Materials and Methods

### 2.1. Study Population

This observational retrospective study included all patients aged 18 or above with ESKD who underwent surgical procedures to create vascular access for dialysis at the Vascular Surgery Department of Targu Mureș County Emergency Hospital in Romania. The study period was from January 2020 to December 2022. We excluded patients hospitalized due to an existing AVF dysfunction, patients with hematological diseases, autoimmune diseases, signs of infections, and peripheral arterial disease stage IV Leriche Fontaine. Additionally, due to the COVID-19 pandemic period, during which this study was conducted, we excluded patients who were known to have been infected with COVID-19 prior to hospitalization. Finally, we excluded patients whose surgical creation of the AVF was unsuccessful or did not have a permeable AVF at discharge. Further, the 116 patients enrolled in this study were divided into two groups based on their preference for hospitalization: *out-patients* (the patients were discharged on the same day, two hours after the surgical intervention) and *in-patients* (the patients stayed overnight in the hospital and were discharged on the second day after their surgery). We obtained the patient demographics, comorbidities, and pre-operative laboratory data from the hospital’s electronic database. We also recorded the type of AVF performed and the dominant or non-dominant limb where the vascular access was performed. Given the lack of benefit of systemic heparinization in terms of long-term patency and the increased risk of post-operative hemorrhagic complications [28,29,30], no patient received heparin during or after the surgical procedure of AVF creation. Furthermore, we recorded the expenses for laboratory analyses, surgical consumables, hospitalization, and total patient costs, respectively, all of which are expressed in EUR.

### 2.2. Comorbidities and Laboratory Data

For each patient, we documented their cardiovascular pathologies, including arterial hypertension, ischemic heart disease, atrial fibrillation, and peripheral arterial disease (PAD). We also noted their medical history of diabetes mellitus (DM), malignancy, prevalent myocardial infarction (MI), prevalent stroke, obesity, and active smoking. 

Upon hospitalization, blood samples were collected from each patient to analyze their blood count, biochemistry, and ionogram. We measured the levels of neutrophils, monocytes, platelets, and lymphocytes and calculated three biomarkers to monitor the difference in systemic inflammatory status between the two groups of patients. Thus, we calculated the following three biomarkers: neutrophil-to-lymphocyte ratio (NLR), monocyte-to-lymphocyte ratio (MLR), and platelet-to-lymphocyte ratio (PLR), due to their availability and prognostic role in poor outcomes of various cardiovascular diseases, as well as in mortality and vascular access dysfunction in ESKD patients [31,32,33,34]. Additionally, we measured the levels of white blood cells, hemoglobin, hematocrit, glucose, potassium, sodium, creatinine, and blood urea nitrogen (BUN).

### 2.3. Study Outcomes

The primary endpoint of this study was to assess the cost-effectiveness of surgically creating an AVF in an ambulatory vs. in-hospital setting by comparing the costs required for the two types of admissions. As a secondary endpoint, we examined the clinical implications of the two types of admissions mentioned earlier. We monitored the rate of post-operative complications such as bleeding, hematoma, signs of local infection, and wound dehiscence during the first two weeks after the surgical intervention. We also observed the maturation rate of the AVF six weeks after the surgery and the long-term primary patency. The criteria for the maturation of the AVF were based on the guidelines recommended by the ESVS [9] and the National Kidney Foundation’s Kidney Disease Outcomes Quality Initiative (KDOQI) [35]. These guidelines state that the vein should have a minimum diameter of 6 mm and be less than 6 mm deep compared to the skin [9,35]. Additionally, the AVF should have a flow rate of at least 600 mL/min [9,35]. The primary patency status of the AVF was obtained from the chronic dialysis centers, and it was defined as the inability to perform hemodialysis at the vascular access level. The status of the patients’ long-term AVF was monitored until vascular access dysfunction was reported or until 31 December 2023.

### 2.4. Statistical Analysis

The statistical analysis for this study was performed with SPSS for Mac OS, version 29.0.1.1 (SPSS, Inc., Chicago, IL, USA). The continuous variables are presented as mean ± standard deviation (SD) values or median (quartile 1–quartile 3). To compare the characteristics between groups, we used the chi-square test for the dichotomous variables, and for the continuous variables, we used the Mann–Whitney U test and Student’s *t*-test. Kaplan–Meier curves were used to model the crude association between the type of hospitalization and long-term AVF primary patency failure. The log-rank test was used to compare the curves. We conducted a multivariate linear regression analysis to determine if the type of hospitalization or other variables can predict local complications or the maturation failure of AVF. Furthermore, we utilized multivariate Cox proportional hazard analysis to identify the independent predictors of long-term primary patency failure of AVF. We conducted a detailed statistical analysis using different adjustment models. In addition to the unadjusted model, we created three more models: model 2, which included age and sex; model 3, which included age, sex, and cardiovascular risk factors (hypertension, diabetes mellitus, history of myocardial infarction, peripheral arterial disease, smoking, obesity); and model 4, which included age, sex, cardiovascular risk factors (hypertension, diabetes mellitus, history of myocardial infarction, peripheral arterial disease, smoking, obesity), and malignancy. All tests were two-tailed, and a *p*-value less than 0.05 was considered statistically significant.

## 3. Results

In this study, we recruited 116 patients with ESKD who had a permeable AVF during their discharge. The average age of these patients was 61.98 years, and 56.03% were male. The most prevalent comorbidity was hypertension (89.66%), followed by ischemic heart disease (65.52%) and diabetes (41.38%) (Table 1). Regarding the in-patient comorbidities, there was a higher prevalence of PAD (24.32% vs. 6.33%, *p* = 0.006), malignancy (16.22% vs. 3.80%, *p* = 0.020), and previous MI (24.32% vs. 8.86%, *p* = 0.012). In addition, active smoking (32.43% vs. 11.39%, *p* = 0.006) and obesity (37.83% vs. 17.72%, *p* = 0.018) were more frequent among these patients. Therefore, we observed that the patients who preferred in-hospital admission had a higher incidence of comorbidities.

At the blood panel analysis, the in-patients had lower levels of WBC (6.92 vs. 8.12, *p* = 0.004), neutrophils (4.55 vs. 5.39, *p* = 0.025), lymphocytes (1.21 vs. 1.52, *p* = 0.034), and monocytes (0.51 vs. 0.59, *p* = 0.032). However, there were no differences between the two groups regarding the systemic inflammatory biomarkers: NLR (*p* = 0.838), MLR (*p* = 0.926), and PLR (*p* = 0.265), as well as the AVF type: RC-AVF (*p* = 0.245), BC-AVF (*p* = 0.185), and BB-AVF (*p* = 0.898). Additionally, we did not register any difference between the two groups of patients regarding the outcomes: local complications (*p* = 0.588), maturation failure (*p* = 0.267), and primary patency (*p* = 0.834). A significant limitation for out-patients is the inability to calculate the diagnosis-related groups (DRGs), which have become the most common mode of hospital payment in the industrialized world in the past decade [36].

**Figure 1:**
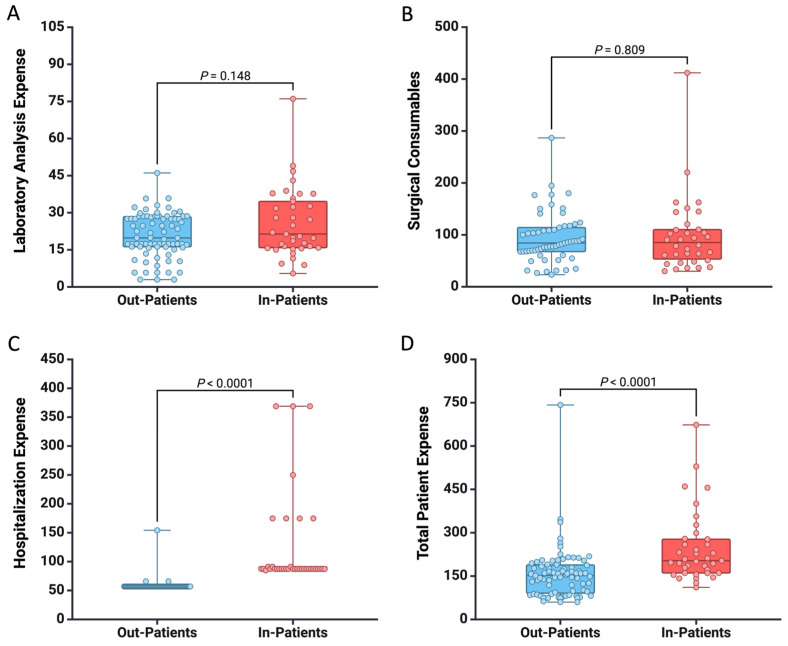
Box plot showing the difference between the two groups regarding the expense for (**A**) laboratory analysis, (**B**) surgical consumables, (**C**) hospitalization, and (**D**) total patient cost. All the values are presented in EUR.

We analyzed the costs incurred by patients based on the type of hospitalization they chose. We found that there was no significant difference in the expenses for laboratory analyses (EUR 21.37 vs. EUR 19.83, *p* = 0.148) and surgical consumables and interventions (EUR 85.15 vs. EUR 84.28, *p* = 0.809) between in-patients and out-patients (Figure 1). However, the patients who chose in-hospital settings and required an overnight stay had higher hospitalization expenses compared to the out-patients (EUR 87.35 vs. EUR 56.86, *p* < 0.0001) (Figure 1). Therefore, overall, the total cost of in-patient care was higher (EUR 203.29 vs. EUR 151.85, *p* < 0.0001) due to additional expenses necessary for patient accommodation.

**Figure 2:**
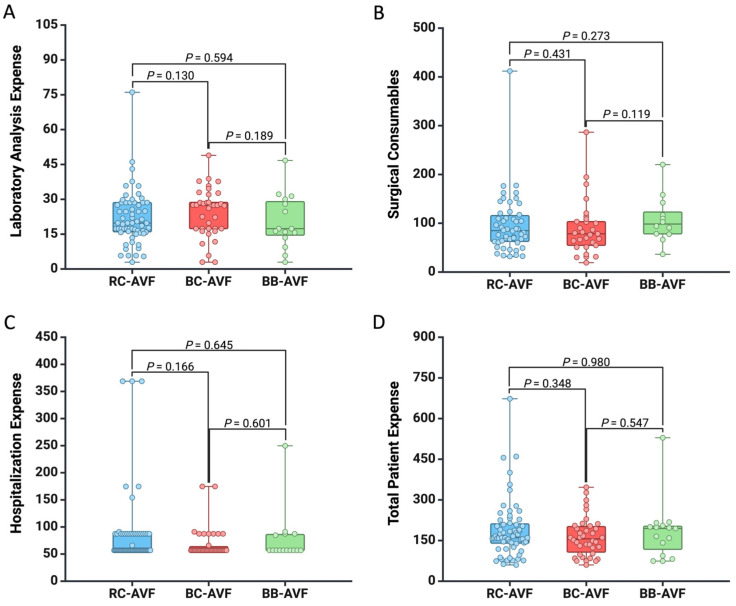
Box plot showing the difference between the AVF type regarding the expense for (**A**) laboratory analysis, (**B**) surgical consumables, (**C**) hospitalization, and (**D**) total patient cost. All the values are presented in EUR.

Further, we thoroughly investigated the cost implications of each type of AVF. We found that there were no significant differences in terms of expenses related to laboratory analysis (all *p* > 0.05), surgical materials (all *p* > 0.05), hospitalization costs (all *p* > 0.05), and total patient expenditure (all *p* > 0.05), as shown in Figure 2.

**Figure 3:**
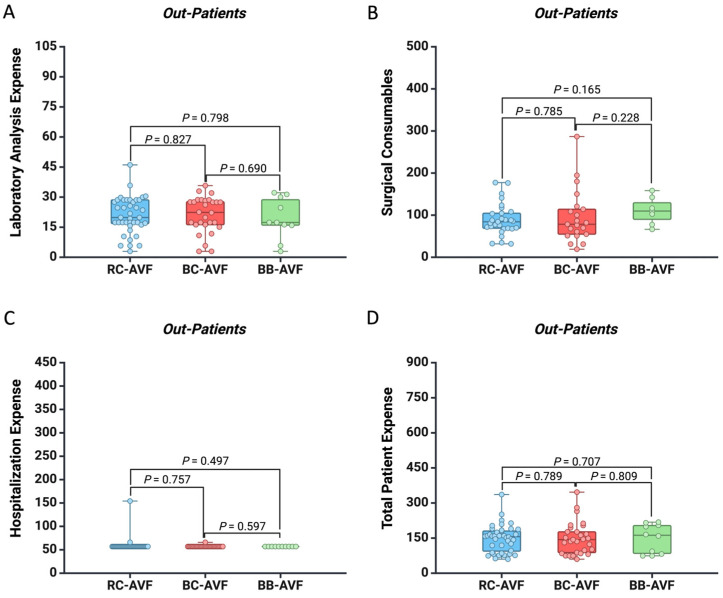
Box plot showing the difference between the AVF type regarding the expense for (**A**) laboratory analysis, (**B**) surgical consumables, (**C**) hospitalization, and (**D**) total patient cost for out-patients. All the values are presented in EUR.

**Figure 4:**
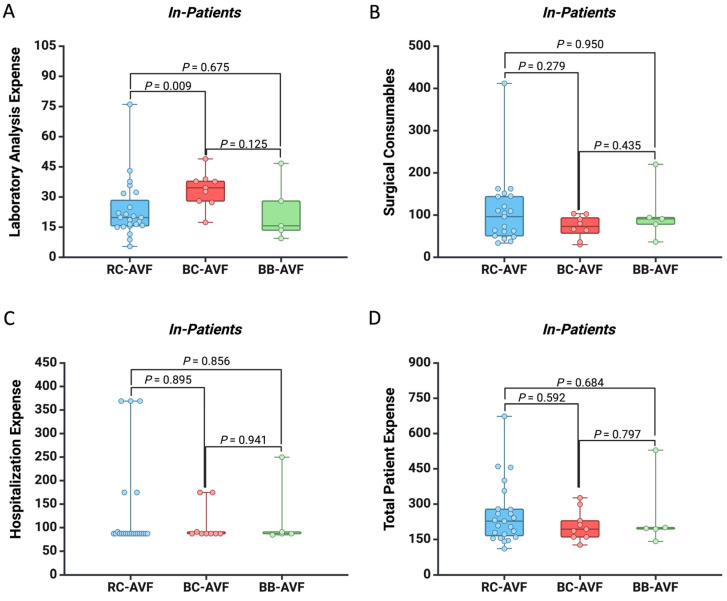
Box plot showing the difference between the AVF type regarding the expense for (**A**) laboratory analysis, (**B**) surgical consumables, (**C**) hospitalization, and (**D**) total patient cost for In-Patients. All the values are presented in EUR.

For a more precise analysis, we separately compared the costs required for each type of AVF for out-patients (Figure 3) and in-patients (Figure 4). We noticed that patients who stayed overnight had a higher expense for laboratory analysis when they had BC-AVF as compared to those with RC-AVF (EUR 33.67 vs. EUR 23.54, *p* = 0.009) (Figure 4). No significant financial difference was observed across AVF types and hospitalization settings.

**Figure 5:**
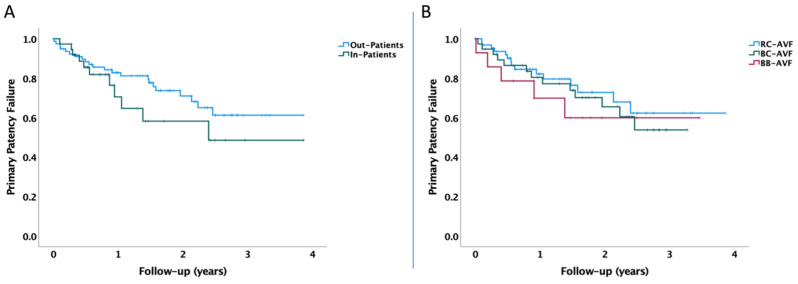
Kaplan–Meier plot of AVF primary patency failure during follow-up, based on (**A**) type of hospitalization chosen and (**B**) type of AVF performed. The *p*-value was calculated with the log-rank test, using pooled over strata for the difference between the out-patients and in-patients, as well as pooled over strata and pairwise over strata for the difference between the type of AVF performed.

In our subsequent analysis, we observed that there was no significant difference between the hospitalization types chosen by patients in terms of AVF primary patency failure, as shown in Figure 5A (*p* = 0.195). Furthermore, the type of AVF performed did not have an impact on primary patency failure during the follow-up (*p* = 0.529, pooled over strata), as shown in Figure 5B.

Furthermore, with the help of multivariate linear regression and a multivariate Cox proportional hazard analysis, we analyzed the relationship between the hospitalization type and local complications, 6-week maturation rate, and long-term primary patency failure. We found no significant association between the hospitalization type and the recorded outcomes (*p* = 0.590 for local complication incidence, *p* = 0.274 for AVF maturation failure, and *p* = 0.199 for AVF primary patency failure) (Table 2). However, due to the presence of a greater number of comorbidities and risk factors in the in-patient group, we chose to analyze three additional adjustment models. Thus, in model 2, we adjusted for sex and age; in model 3, for sex, age, and cardiovascular risk factors; and in model 4, for all previously mentioned variables, and additionally for malignancy. As shown in Table 2, we did not observe any differences between the two types of admissions, regardless of age, sex, cardiovascular risk factors, or malignancy.

## 4. Discussion

In this study, we investigated the cost-effectiveness and clinical implications of creating AVF surgically in either an ambulatory or in-hospital setting. We found no difference in post-operative functionality or clinical implications between the two hospitalization types. Furthermore, we observed no difference in the costs required for laboratory analysis and surgical consumables related to the AVF creation between the two patient groups. However, due to the expenses associated with accommodating in-patients, the final cost per patient was higher for the in-patients than for the out-patients (*p* < 0.0001).

Recently published studies analyzing the feasibility of performing surgical or endovascular interventions in an ambulatory setting have demonstrated a low incidence of post-operative complications [20,23,37,38]. According to Margulis et al. [23], the out-patients had a lower rate of re-admissions (*p* = 0.0386) one week after surgery compared to the in-patients. The researchers followed post-operative complications up to 30 days after the index events. In a study conducted by Mestres et al. [37] involving 1012 patients with ESKD, there were no significant statistical differences observed with respect to the incidence of reinterventions, significant infection, bleeding or hematoma, and 24 h post-operative death between the ambulatory and non-ambulatory cases. Jiménez-Almonacid et al. [20] showed that ambulatory AVF surgery is feasible in case of vascular access dysfunction or emergency surgery.

Several factors can cause maturation failure and AVF dysfunction in the long term, including diabetes [13], female sex [39,40], weight [39], increased systemic inflammation [12], and pre-operative vessel characteristics. Additionally, the timing, type, and cannulation time of vascular access play a crucial role in the evolution of vascular access for dialysis [41,42,43,44,45,46,47]. A study conducted by Jeong et al. [41] on 524 patients with ESKD revealed that the patients who underwent HD through an AVF had a higher overall survival rate (*p* = 0.013), with no significant difference in primary and secondary AVF patency rates at the Kaplan–Meier survival analysis. However, studies by Hiremath et al. [42], Hod et al. [43], and Shechter et al. [44] concluded that the early creation of an AVF is not optimal for elderly patients. In addition to the previously presented study results, Rayner et al. [45] conducted a multicenter study on a cohort of 3674 patients. The authors showed that patients with prior temporary access had a higher incidence of AVF failure (RR: 1.81, *p* = 0.01). Additionally, they found a higher prevalence of AVF dysfunction when cannulation was performed within 14 days of AVF creation (*p* = 0.006).

Hospitals use DRG as a method for reimbursement of in-patient services, which in the case of vascular surgery is more affected by post-operative care, the duration of the patient’s hospital stay, and coding errors, rather than the surgical procedure itself [48,49,50,51,52,53]. For instance, Voicu et al. [48] found that longer length of stay, and pre-operative dialysis are associated with negative margins in the multivariate regression. Similarly, Ayub et al. [52] discovered that incorrect coding of the complexity of elective endovascular aneurysm repair led to a significant loss of billing opportunity and recommended reconsidering coding practices. Perri et al. [53] registered a decrease in reimbursement from 2010 to 2015.

The current study has some limitations that should be mentioned. Firstly, this study was conducted retrospectively and had a relatively small group of patients from a single center, where endovascular AVF was not performed. Secondly, each patient decided on the type of hospitalization. Therefore, future randomized clinical trials are needed to improve the accuracy of the results. Another limitation was the lack of pre-operative ultrasound features and the severity of intimal hyperplasia in the long term. Thirdly, given this study’s retrospective nature, we could not quantify each patient’s health-related quality of life and its impact on the incidence of local complications, the maturation rate, and long-term primary patency. Lastly, another limitation is the lack of inclusion of information related to the use and dosage of antiplatelet drugs for the entire cohort in the statistical analysis, as this information was not available for all enrolled patients in the hospital’s electronic database.

## 5. Conclusions

In conclusion, there are no significant differences in the clinical implications and short-term and long-term complications of AVF between *out-patient* and *in-patient* admissions. Additionally, we found no variation in the costs associated with laboratory tests and surgical supplies for AVF creation. Therefore, it is safe to perform ambulatory AVFs, which can reduce the risk of hospital-acquired infections and provide greater comfort to the patient.

## Figures and Tables

**Table 1:** Demographic data, comorbidities, risk factors, laboratory data, type of AVF, and outcomes analyzed in this study were presented comparatively, according to the patients’ hospitalization type.

Variables	All Patients *n* = 116	Out-Patients *n* = 79	In-Patients *n* = 37	*p*-Value
Age mean ± SD	61.98 ± 14.3	60.98 ± 14.34	64.10 ± 14.16	0.274
Male sex no. (%)	65 (56.03%)	43 (54.43%)	22 (59.46%)	0.611
Comorbidities and risk factors, no. (%)				
Arterial Hypertension	104 (89.66%)	70 (88.61%)	34 (91.89%)	0.588
Ischemic Heart Disease	76 (65.52%)	50(63.29%)	26 (70.27%)	0.461
Atrial Fibrillation	13 (11.21%)	6 (8.86%)	6 (16.22%)	0.242
Diabetes Mellitus	48 (41.38%)	32 (40.51%)	13 (43.24%)	0.780
Peripheral Arterial Disease	14 (12.07%)	5 (6.33%)	9 (24.32%)	***0.006***
Malignancy	9 (7.76%)	3 (3.80%)	6 (16.22%)	***0.020***
History of Myocardial Infarction	15 (12.93%)	6 (8.86%)	9 (24.32%)	***0.012***
History of Stroke	8 (6.90%)	4 (5.06%)	4 (10.81%)	0.255
Active Smoking	21 (18.10%)	9 (11.39%)	12 (32.43%)	***0.006***
Obesity	28 (24.13%)	14 (17.72%)	14 (37.83%)	***0.018***
Laboratory data, median (Q1–Q3)				
WBC	7.83 (6.16–9.42)	8.12 (6.65–9.67)	6.92 (5.38–8.21)	***0.004***
Potassium mmol/L	5.16 (4.65–5.64)	5.19 (4.79–5.69)	4.99 (4.55–5.51)	0.282
Sodium mmol/L	139.6 (138–141)	140 (138–141)	139 (137–140)	0.269
Glucose (mg/dL)	101.5 (89–129.75)	101 (88.8–131.95)	102 (91.7–128.25)	0.914
BUN (mg/dL)	121.2 (89.9–161.6)	118.65 (89.45–161.1)	124.12 (97.05–157.29)	0.504
Creatinine (mg/dL)	6.32 (5.04–8.04)	6.35 (5.23–7.70)	5.86 (4.43–9.27)	0.826
Hemoglobin g/dL	10.6 (9.34–11.6)	10.6 (9.11–11.52)	10.3 (9.57–11.92)	0.761
Hematocrit %	32.5 (28.76–35.75)	32.8 (28.45–35.8)	32.4 (30–35.62)	0.629
Neutrophils ×10^3^/μL	5.11 (4.07–6.35)	5.39 (4.31–6.98)	4.55 (3.77–5.70)	***0.025***
Lymphocytes ×10^3^/μL	1.44 (1.07–1.95)	1.52 (1.16–1.99)	1.21 (0.92–1.79)	***0.034***
Monocyte ×10^3^/μL	0.577 (0.44–0.76)	0.59 (0.48–0.77)	0.51 (0.4–0.72)	***0.032***
PLT ×10^3^/μL	217 (181–282.5)	225 (188.5–290.4)	199.55 (169.75–262.5)	0.157
NLR	3.33 (2.41–4.60)	3.33 (2.45–4.72)	3.35 (2.39–4.55)	0.838
MLR	0.39 (0.28–0.50)	0.39 (0.27–0.54)	0.40 (0.30–0.46)	0.926
PLR	145.33 (108.33–193.89)	140.59 (106.64–191.98)	158.38 (126.28–196.73)	0.265
AVF type and placement, no. (%)				
RC-AVF	63 (54.31%)	40 (50.63%)	23 (62.16%)	0.245
BC-AVF	38 (32.76%)	29 (36.71%)	9 (24.32%)	0.185
BB-AVF	15 (12.93%)	10 (12.66%)	5 (13.51%)	0.898
Dominant Limb	23 (19.83%)	15 (18.99%)	8 (21.62%)	0.740
Non-Dominant Limb	93 (80.17%)	64 (81.01%)	29 (78.28%)	
Outcomes				
Local Complication, no. (%)	12 (10.34%)	9 (11.39%)	3 (8.11%)	0.588
Maturation Failure, no. (%)	19 (16.38%)	15 (18.99%)	4 (10.81%)	0.267
Primary Patency, no. (%)	83 (71.55%)	57 (72.15%)	26 (70.27%)	0.834
Follow-Up Period (years) mean ± SD	1.39 ± 0.93	1.44 ± 0.98	1.29 ± 0.82	***<0.001***
Diagnosis-Related-Group, mean ± SD	4.63 ± 0.98	-	4.63 ± 0.98	-

White blood cells (WBC), blood urea nitrogen (BUN), platelets (PLT), neutrophil-to-lymphocyte ratio (NLR), monocyte-to-lymphocyte ratio (MLR), platelet-to-lymphocyte ratio (PLR), arteriovenous fistula (AVF), radiocephalic arteriovenous fistula (RC-AVF), brachiocephalic arteriovenous fistula (BC-AVF), brachiobasilic arteriovenous fistula (BB-AVF).

**Table 2:** The association between the type of hospitalization and post-operative events, such as local complications, 6-week AVF maturation failure, and long-term AVF primary patency failure.

		**Local Complications**		
		**OR**	**95% CI**	***p*-Value**
In-Patients vs. Out-Patients	Model 1	0.69	0.17–2.67	0.590
	Model 2	0.69	0.17–2.76	0.609
	Model 3	0.75	0.17–3.34	0.708
	Model 4	0.86	0.19–3.93	0.848
		**AVF Maturation Failure**		
		**OR**	**95% CI**	***p*-Value**
In-Patients vs. Out-Patients	Model 1	0.52	0.16–1.68	0.274
	Model 2	0.53	0.17–1.76	0.303
	Model 3	0.55	0.17–1.85	0.337
	Model 4	0.59	0.18–1.98	0.395
		**AVF Primary Patency Failure**		
		**HR**	**95% CI**	***p*-Value**
In-Patients vs. Out-Patients	Model 1	1.62	0.77–3.39	0.199
	Model 2	1.11	0.52–2.35	0.800
	Model 3	1.08	0.48–2.43	0.850
	Model 4	1.20	0.53–2.72	0.662

Model 1: unadjusted; model 2: age and sex; model 3: age, sex, cardiovascular risk factors (hypertension, diabetes mellitus, history of myocardial infarction, peripheral arterial disease, smoking, obesity); model 4: age, sex, cardiovascular risk factors (hypertension, diabetes mellitus, history of myocardial infarction, peripheral arterial disease, smoking, obesity), and malignancy.

## Data Availability

The data that support the findings of this study are available from the corresponding author upon reasonable request.
